# Topological photonics for single-photon sources

**DOI:** 10.1038/s41377-025-02068-6

**Published:** 2026-01-01

**Authors:** Fei Ding

**Affiliations:** Institut für Festkörperphysik, Leibniz Universität Hannover, Appelstraße 2, 30167 Hannover, Germany

**Keywords:** Single photons and quantum effects, Quantum optics

## Abstract

The pursuit of high-quality single-photon sources has long been hampered by challenges in improving the performance and robustness. While traditional microcavity structures can achieve impressive performance, they suffer from extreme sensitivity to manufacturing uncertainty, structural disorders, and scatterings. Topological photonics potentially offers a powerful toolbox for solving these problems. A recent breakthrough by researchers from the Beijing Academy of Quantum Information Sciences, published in *Light: Science & Applications*, exploits a topological bulk state rather than the already reported edge and corner states to enhance the single photon emission for a quantum dot.

A recent study suggests that topological photonics^1^ may offer a promising route to addressing a key challenge in the development of solid-state single-photon sources^2^. Quantum dot (QD) single-photon sources have evolved from early micropillar cavities^3^ and photonic crystal structures^4^ to today’s popular circular Bragg gratings^5^. However, these “conventional” designs share several common challenges. They demand extreme manufacturing precision, which drives up costs and reduces yields. The random distribution of embedded QDs necessitates complex positioning techniques^6^ to achieve effective spatial coupling with cavity modes, not even to mention their spectral coupling. Therefore, they are not robust yet for practical applications^7^.

Recent innovations, such as tunable Fabry-Pérot microcavities^8,9^, have addressed specific limitations but have not fundamentally solved the robustness problem. This is where topological photonics offers a potential solution^10^. Unlike conventional designs that rely on precise geometric control^11^, topological protection provides intrinsic immunity to structural disorders and scattering losses, which could be a game-changing advantage for practical quantum photonic devices.

The key innovation of the work by Mao et al. lies in exploiting “band-inversion-induced reflection” to confine bulk states laterally^2^. By interfacing two photonic crystals with opposite topological properties (the trivial and non-trivial regions), see Fig. 1a, light waves near the Γ point experience reflection at the topological boundary, creating effective confinement without relying on traditional geometric resonance mechanisms. This approach was first employed in developing topological bulk lasers^12,13^ and it yields two crucial advantages. First, the confinement occurs near the Γ point in momentum space, naturally producing vertical emission with minimal divergence. This is ideal for efficient photon extraction into optical fibers. Second, the bulk nature of these states (topologically protected) makes them inherently robust against edge/corner irregularities, see for example the “Q”-shaped irregularity in Fig. 1b. Mao et al. proved the potential of bulk topological states in quantum photonics. They designed and demonstrated a single-photon source by coupling a single InAs/GaAs QD to a topological bulk cavity, see Fig. 1c.

**Fig. 1:**
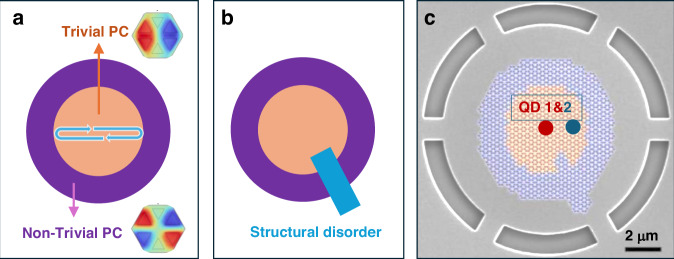
Design of the topological photonic cavity with embedded quantum dots. **a** Design of the topological bulk cavity. The trivial and non-trivial photonic crystal (PC) regions possess opposite modal parities (insets, adapted from Ref. 2 with permission), leading to the band-inversion-induced reflection (indicated by the arrows) and creating an effective in-plane confinement of light. **b** To prove the robustness, a structural disorder/irregularity was introduced. **c** SEM image of a fabricated ‘Q’-shaped topological bulk cavity patterned on a GaAs wafer. The cavity is composed of a trivial PC (orange) encircled by a topological PC (purple). Two different QDs were investigated. Adapted from Ref. 2 with permission

Besides the robustness, a notable advantage of this design is avoiding the use of precise QD positioning, see Fig. 1c. Traditional high-Q cavities require QDs to be positioned within tens of nanometers of field maxima, a low-probability, high-cost endeavor^5,6^. In contrast, topological bulk cavities, with their moderate Q factors (~100) and extended mode areas, can achieve Purcell factors above 1.6 across 2.5 μm^2^ areas. This dramatically increases coupling probabilities while reducing manufacturing complexity. In Fig. 1c, two different QDs at different locations were investigated, and their radiation properties were similarly modulated by the topological cavities. While the achieved Purcell enhancement factor is modest compared to state-of-the-art cavities, the robustness represents a fundamental trade-off that favors practical applications over fabrication complexity. As discussed before, the vertical directionality of topological bulk state emission is also a highlight. It produces vertical emission with 6.2° divergence, achieving simulated extraction efficiencies up to 92% when integrated with reflectors.

The demonstrated technology builds on mature III–V semiconductor processes, for example, the molecular beam epitaxy, electron beam lithography, and etching. This ensures compatibility with existing industrial fabrication infrastructure. The irregular cavity tolerance would relax the constraints in semiconductor production processes. The planar geometry facilitates integration with electronic contacts, potentially enabling electrically tunable sources without the complex bridging structures required by circular Bragg gratings^14^.

The combination of topological photonics and semiconductor quantum light sources has witnessed remarkable progress in recent years, with the current study reporting the topological bulk states for single photon extractions. In this context, the intrinsic robustness afforded by topological protection may prove equally valuable in achieving record-breaking performances or discovering new physics. This would distinguish the topological approaches from many conventional optical microcavity platforms.

Looking ahead, several challenges must be addressed in this emerging field of “topological quantum photonics”. The single-photon sources performance in recent similar works is still moderate^15–17^, indicating substantial room for improvement through further optimization of photonics design and material engineering. The transition from individual components to a full-fledged topological quantum photonic platform presents significant integration challenges. Such a platform would feature, for example, arrays of spatially and spectrally multiplexed topological single-photon and entangled-photon sources, on-chip waveguides, routers, and detectors. The challenges would lie in, just name a few here, minimizing inter-component losses, managing spectral matching, ensuring temporal synchronization, and possibly, maintaining the topological protection throughout the integrated system.
